# Effects of Long-Term Endurance Exercise on Cardiac Morphology, Function, and Injury Indicators among Amateur Marathon Runners

**DOI:** 10.3390/ijerph20032600

**Published:** 2023-01-31

**Authors:** Jianzhong Hu, Songqing Zhou, Suryeon Ryu, Kaitlyn Adams, Zan Gao

**Affiliations:** 1 School of Physical Education, Hengyang Normal University, Hengyang 421002, China; 2 School of Kinesiology, University of Minnesota, Minneapolis, MN 55455, USA

**Keywords:** heart volume, high-intensity endurance exercise, myocardial injury, stroke volume

## Abstract

The purpose of this study was to investigate the effects of long-term endurance exercise on cardiac morphology and function, as well as injury indicators, among amateur marathon runners. We recruited 33 amateur runners who participated in a marathon. Participants were divided into experimental and control groups according to their National Athletic Grade. The experimental group included participants with a National Athletic Grade of 2 or better, and the control group included participants who did not have a National Athletic Grade. Cardiac morphology, function, and injury indicators were assessed before and after the participants’ involvement in the Changsha International Marathon. All cardiac morphology and function indicators returned to pre-race levels at 24 h post-race, and left ventricular end-diastolic volume and left ventricular end-systolic volume indicators showed similar trends. Both stroke volume (SV) and percent fractional shortening (%FS) indicators showed similar trends in changes in the measurements before and after the race. SV showed no change between the pre-race and post-race periods. On the other hand, %FS showed a significant increase in the immediate post-race period, followed by restoration of its level at 24 h post-race. Among myocardial injury indicators, serum levels of cardiac troponin I, creatine kinase (CK), creatine kinase-MB (CK-MB), lactate dehydrogenase (LDH), aspartate aminotransferase (AST), and N-terminal pro-b-type natriuretic peptide (NT-proBNP) measured before the race, immediately after the race, and 24 h after the race displayed similar trends in changes among CK, CK-MB, LDH, and AST, while NT-proBNP levels did not change. We concluded that high-level amateur marathon runners had greater heart volumes, as well as wall and septal thicknesses, than low-level marathon runners, with differences in heart volume being the most pronounced. Long-term high-intensity endurance exercise caused some damage to the hearts of amateur runners. High-level runners showed better myocardial repair ability, and their levels of myocardial injury markers showed greater decreases at 24 h post-race, while low-level runners had poorer myocardial repair ability.

## 1. Introduction

In recent years, the number of people participating in endurance training, competitive long-distance endurance events, and high-intensity interval training has significantly increased worldwide [1]. However, with the increasing popularity of endurance and ultra-endurance sports programs, participants older than 35 years of age have become more susceptible to cumulative heart injury [2,3]. Although current physical activity guidelines suggest significant health benefits from 150 min of moderate exercise or 75 min of vigorous exercise per week, endurance athletes typically exercise 15 to 20 times more than the amount recommended by these guidelines [4,5]. This is problematic because excessive endurance exercise can lead to increased risks of atrial fibrillation (AF) and atrial flutter [6]. Moreover, vigorous-intensity exercises can be associated with cardiac maladaptation, including accelerated coronary artery calcification, exercise-induced release of cardiac biomarkers, myocardial fibrosis, and right ventricular dilation and dysfunction [7,8]. Additionally, these exercises are associated with reduced left ventricular compliance [9].

Exercise-induced cardiac remodeling (EIRC) is the process by which long-term participation in exercise training results in significant changes in the structure and function of the heart [10]. This process is considered a physiological adaptation of the heart to exercise stimuli and is fundamentally different from cardiac remodeling caused by myocardial disease [11]. Exercise cardiac remodeling was first believed to primarily involve left ventricular hypertrophy, including concentric and eccentric hypertrophy, accompanied by changes in cardiac systolic and diastolic function [12]. As research advanced, Weiner and Baggish reported exercise hypertrophy in the right ventricle [13]. Although long-term endurance exercise is believed to cause cardiac remodeling, quantitative research regarding its associated long-term effects is lacking.

The indicators of cardiac injury and overload for different exercise types, durations, and intensities remain controversial. For instance, researchers found that following high-intensity exercise, the levels of myocardial injury indicators, including creatine kinase (CK), myoglobin (Myo), and cardiac troponin (cTnT), were elevated in professional athletes [14,15,16,17] and were restored to varying degrees after competition [17] contrary to clinical cut-offs. Current investigations for high-intensity exercise mainly focus on laboratory examinations of professional athletes or of the general population on psychological impacts on participants. However, attention to natural post-race cardiac function in amateur marathon runners is limited.

Thus, this study aimed to provide a theoretical basis to guide the general public on sports participation. Specifically, this study examined changes in cardiac morphology and function, as well as myocardial injury indicators, among amateur marathon runners in China before and after participation in a race. In addition, we explored the preliminary effects of long-term high-intensity endurance exercise on cardiac function in nonprofessional athletes without the psychological influence of the laboratory environment. The findings of this study have the potential to help athletes improve their cardiac functions and prevent unnecessary injuries during practice.

## 2. Materials and Methods

### 2.1. Study Design and Procedures

This study employed a causal comparative study design, where participants were allocated to either experimental or control groups based on their National Athletic Grade. In detail, the marathon time for Grade 1 athletes is 2 h and 32 min, the time for Grade 2 athletes is 2 h and 53 min, and the time for Grade 3 athletes is 3 h and 2 min [18]. Participants were divided into experimental and control groups based on their National Athletic Grade. In detail, participants who were Grade 2 or better (i.e., Grade 1) were allocated to the experimental group, and those who did not meet the National Athletic Grade (i.e., lower than National Athletic Grade 3) were allocated to the control group. The study procedures were approved by the university’s ethics committee at Hengyang Normal University (no.:180809P09), and written informed consent was obtained from all participants before data collection. All anthropometric data were collected by the researchers in a laboratory setting. Echocardiography and blood sample data were also collected in a highly controlled laboratory. The relevant procedures were performed in strict accordance with the reagent and instrument operating instructions.

### 2.2. Research Participants

To detect a mean difference in primary outcomes between the experiment and control groups with 80% power and 5% significance, assuming a standard deviation of 10, this project required 15 subjects per group. This study recruited amateur marathon runners who met the following inclusion criteria: (1) had participated in and completed at least one international marathon; (2) were aged from 18 to 50 years of age; (3) were male amateur runners; (4) had no self-reported or diagnosed physical or mental disability; and (5) were willing to provide informed consent to participate in the study. The exclusion criteria for participation in this study were: (1) physical disabilities that limited his or her engagement in physical activities; (2) mental conditions that limited his or her engagement in physical activities; and (3) declined completion of the informed consent form. Due to the influence of subjective and objective factors, such as epidemics and offsite conditions, a total of 33 participants completed the study, including 15 participants from the experimental group and 18 participants from the control group. Signed informed consent was provided prior to the start of the experiment. The sample descriptive data are shown in Table 1.

There was a particular rationale for the age range of participants investigated in this study. In detail, we obtained the performance data of amateur marathon runners in China between 2014 and 2017, with data taken from the website RunChina (www.runchinamarathon.com, accessed on 22 January 2023) [19]. The average performance of the different age groups in the same year showed no differences in the decline of performance in the 35–39, 40–44, and 45–49 year age groups, regardless of sex, compared to the performance of the 18–34 year age group for average performance in all races in all years from 2014 to 2017 (*p* > 0.05). The decreased performance in the other age groups showed greater differences with increasing age. Among the functions involved in the maintenance of health, cardiovascular function plateaued during the decline: runners aged 35–49 years showed a slow decline, while those over 49 years of age showed a trend of significant decline with increasing age every 5 years. Therefore, in this study, we recruited only adults aged from 18 to 50 years to investigate the effects of marathon exercise on the cardiovascular system.

### 2.3. Measures

#### 2.3.1. Anthropometric and Demographic Information

The participants’ height and weight were measured to calculate their body mass index (BMI; kg/m^2^). Height was assessed using a Seca stadiometer (Seca, Hamburg, Germany). Weight was measured to the nearest 10th of a kilogram using a Tanita BC-558 IRONMAN^®^ Segmental Body Composition Monitor (Tanita, Tokyo, Japan).

In addition, participants’ age and general information about their training (i.e., average weekly training distance, duration of long-distance training) and past marathon experiences (i.e., number of participations, most recent completion time) were self-reported.

#### 2.3.2. Echocardiography

Echocardiography was performed with the participants at rest in the lateral decubitus position, with a probe placed between the third and fourth intercostal space of the left sternal margin [20]. The electrocardiogram readings were recorded simultaneously to determine diastolic and systolic phases. All echocardiographic data were stored and analyzed with respect to the current standardized measurements and analysis in China. The left ventricular morphological indicators included: (1) left ventricular end-diastolic volume (EDV); (2) left ventricular end-systolic volume (ESV); (3) interventricular septum thickness in diastole (IVSD); (4) interventricular septum thickness in systole (IVSS); (5) left ventricular posterior wall thickness in diastole (LVPWD); (6) left ventricular posterior wall thickness in systole (LVPWS); and (7) left ventricular posterior wall motion (LVPWM). The higher the values of the aforementioned outcomes, the better the cardiac morphological indicators would be. The left ventricular function indicators were fractional shortening (FS), ejection fraction (EF), stroke volume (SV), and cardiac output (CO) [21,22]. The higher the values of FS, EF, SV, and Co, the better the cardiac function would be.

#### 2.3.3. Blood Markers

Blood was drawn intravenously to measure levels of cardiac troponin I (cTnI), creatine kinase (CK), creatine kinase-MB (CK-MB) isoenzyme, lactate dehydrogenase (LDH), aspartate aminotransferase (AST), and N-terminal pro-b-type natriuretic peptide (NT-proBNP). Phlebotomy was performed by a lab technician. The technician held the participant’s middle or ring finger in an upward position and then lanced the palm-side surface of the finger. The technician pressed firmly on the finger when making the puncture. These blood outcomes were the cardiac injury indicators. The lower the values of the abovementioned outcomes, the better the cardiac injury indicators would be.

### 2.4. Statistical Analysis

All data were processed using IBM SPSS Statistics for Windows (Version 21.0, Armonk, NY, USA). First, descriptive statistics were used to describe the participants’ demographic outcomes (i.e., age and sex) and BMI, as well as means and standard deviations of the study outcome variables. One-way repeated measures analysis of variance (ANOVA) was used, with data expressed as mean ± standard deviation. A *p* < 0.05 was defined as a significant difference, and *p* < 0.01 as a highly significant difference.

## 3. Results

### 3.1. Descriptive Analysis

The participants’ demographic information, basic indicators of physical fitness, and exercise training conditions are shown in Table 1.

The data in Table 1 suggest that participants in the experimental group trained for longer distances each week and had participated in more marathons compared to the control group (*p* < 0.05). Participants in the experimental group also had shorter race completion times compared to the control group for the most recent marathon they participated in (*p* < 0.05). The findings were consistent with the levels of each group.

### 3.2. Cardiac Morphology and Functional Indicators in Amateur Marathon Runners

Analysis of cardiac morphological indicators before participation in the marathon race (Table 2) showed significantly higher EDV, ESV, IVSD, and IVSS in the experimental group compared to those in the control group (*p* < 0.01). Furthermore, significant differences in EDV, ESV, and IVSD immediately after the race (*p* < 0.05) and significantly higher EDV, ESV, and IVSS values at 24 h after the race (*p* < 0.01) were observed. Compared to their respective pre-race values, EDV, ESV, and IVSS were highly significantly elevated in the immediate post-race period for those in the control group (*p* < 0.01). In the experimental group, the participants’ EDV, ESV, and LVPWM were significantly elevated in the immediate post-race period (*p* < 0.01).

In both the control and experimental groups, all indicators returned to pre-race levels at 24 h post-race, with EDV and ESV indicating similar trends in changes (i.e., highly significant decreases in the immediate post-race period and a return to pre-race levels at 24 h post-race).

The analysis of cardiac function before and after participating in the marathon (Table 3) showed that the SV in participants in the experimental group was significantly higher than that in participants in the control group in the quiet state before the race (*p* < 0.01). Immediately after the race, participants’ SV, EF, and percent fractional shortening (%FS) values in the experimental group were significantly higher than those in the control group (*p* < 0.01). Participants’ SV, CO, and %FS remained significantly different at 24 h after the race (*p* < 0.05/0.01). Compared to pre-race values, participants’ CO and %FS in the control group were significantly higher (*p* < 0.01), with no differences between pre-race and 24 h post-race levels. In the experimental group, participants’ CO, EF, and %FS were significantly higher than their pre-race values (*p* < 0.05/0.01), while only CO was significantly higher at 24 h post-race (*p* < 0.05).

Among cardiac function indicators, both SV and %FS in the control and experimental groups showed the same trends in changes at the three time points, with SV showing no change before and after the race and %FS showing a significant increase in the immediate post-race period, followed by recovery at 24 h.

### 3.3. Changes in Myocardial Injury Indicators in Amateur Marathon Runners

Our data (Table 4) showed no differences between the experimental and control groups before the race. However, the experimental group participants’ cTnI, CK, CK-MB, LDH, and AST levels were significantly lower during the immediate post-race and 24 h post-race periods (*p* < 0.01). Compared to pre-race values, the cTnI, CK, CK-MB, LDH, and AST values in the immediate post-race and 24 h post-race periods in the control group showed significantly greater increases (*p* < 0.01). Participants from the experimental group showed similar changes in the immediate post-race period as those in the control group, with only CK, CK-MB, LDH, and AST levels significantly increased in the 24 h post-race period compared to pre-race levels (*p* < 0.05/0.01).

Participants’ NT-proBNP levels did not change significantly in either the control or experimental group. The participants’ CK, CK-MB, LDH, and AST levels showed similar changes in trend (i.e., significant elevations immediately and 24 h post-race compared to their pre-race values). The participants’ cTnI was significantly elevated in the control group immediately and 24 h post-race, while the cTnI level returned to pre-race levels by 24 h post-race in the experimental group.

## 4. Discussion

### 4.1. Effects of Long-Term Aerobic Endurance Exercise on Cardiac Morphology and Function in Amateur Athletes

Endurance exercise causes adaptive changes in the heart. Some athletes experience decreased left ventricular function after high-intensity endurance exercise. Exercise type is an essential determinant of exercise myocardial remodeling, as the diastolic internal diameters of the right and left ventricles become larger in endurance athletes due to increased blood flow during exercise. In contrast, the chamber volumes of strength training athletes remain unchanged, while their left ventricular walls become thicker, for unknown reasons [4,23]. Benito et al. [24] studied the effects of 8 or 16 weeks of high-intensity exercise on cardiac remodeling in SD rats and reported that 16 weeks of high-intensity exercise significantly increased left ventricular internal diameter. Maria et al. [25] also reported increased left ventricular internal diameters in Wistar rats after 16 weeks of high- and moderate-intensity training. However, the left ventricular internal diameter in the high-intensity exercise group did not differ from that in the moderate-intensity exercise group. In the present study, both the control and experimental groups had been exercising for many years, as indicated by their average weekly training distances, duration of long-distance training years, number of marathons participated in, and most recent marathon completion time.

EDV, ESV, and SV were significantly higher in the experimental group compared to the control group, both immediately before and after the race and 24 h after the race. Meanwhile, LVPWD and LVPWS did not differ between and within the two groups, indicating differences in cardiac morphology between the groups, whereas the experimental group had greater cardiac contractility, greater stroke volume, and, thus, better exercise performance. Analysis of within-group differences demonstrated similar trends in changes in EDV, ESV, and CO in both groups (i.e., significant increases in the immediate post-race period followed by a return to the pre-race quiet state values at 24 h post-race). However, the absence of pre-race and post-race differences in SV in both the control and experimental groups may be related to exercise adaptations due to long-term endurance exercise.

Exercise-induced cardiac remodeling is usually considered a benign physiological adaptation to exercise and is characterized by an enlargement of the heart chambers and increased left ventricular wall thickness [25,26,27,28]. However, exercise-induced and pathological cardiac remodeling cannot be differentiated [29]. The upper limits of right ventricular diameter in athletes (45 mm in women and 50 mm in men) distinguish between physiological cardiac remodeling and pathological conditions [25]. Endurance athletes involved in high-intensity aerobic training have increased left ventricle and left atrium sizes due to proportional increases in stroke output by overload, which manifests as left ventricular hypertrophy [25]. The results of the current study, which focused on the morphology of the left ventricle, showed an increase only in ventricular cavity volume and not in ventricular wall thickness, suggesting that the hearts of amateur marathon runners develop benign adaptations to long-term high-intensity endurance exercise.

At present, cardiac remodeling caused by physical activity is believed to be associated with a cardiac cross-section in athletes. However, it has not been studied in general adult or elderly populations. Heitmann et al. reported that higher cumulative physical activity levels were associated with increased left atrial size in male exercise participants older than 65, whereas moderate levels of physical activity accumulation were associated with increased left ventricular size in women [30]. Left ventricular volume and mass also increased in sedentary young and middle-aged men and women after 1 year or 10 months of high-intensity endurance training [31]. Likewise, the results of the present study show no differences in the IVSD, IVSS, LVPWD, and LVPWS indices in the calm state between the experimental and control groups after different levels of cumulative exercise training. Changes in LV diastolic and systolic function did not differ between the cumulative physical activity groups.

### 4.2. Effects of Long-Term Aerobic Endurance Exercise on Indicators of Cardiac Injury Indicators in Amateur Athletes

Serum markers of myocardial injury, such as cardiac troponin (cTn), creatine kinase MB (CK-MB), and B-type natriuretic peptide (BNP), augmented by 50% during and after the marathon [14,32,33]. Biochemical markers of myocardial injury, such as cTn, increase in response to high-intensity endurance events, such as marathons. This may possibly be due to a loss of desmosome integrity, allowing one cardiomyocyte to slide toward another [34]. Although this change can return to basal values within one week, excessive high-intensity exercise and repetitive injury over the years can lead to myocardial fibrosis, particularly in the atria and right ventricle, which, in turn, can cause atrial and ventricular arrhythmias. In addition, long-term high-intensity endurance exercise accelerates the “aging” of the heart, including coronary artery calcification, ventricular diastolic dysfunction, and hardening of the aortic walls [35]. However, the significance of the increase in cardiac biochemical marker concentrations after high-intensity endurance exercise remains unclear, although it is thought to be a purely transient and beneficial change in cardiovascular adaptation to high-intensity endurance exercise [36,37].

Heidbüchel et al. [35] suggested that a single bout of high-intensity endurance exercise could cause acute damage to the heart, which was reversible with adequate recovery and repair time. The authors also stated that a healthy “athlete’s heart” (hypertrophic and balanced, with little or no fibrosis) develops over a long period of high-intensity endurance exercise. However, if the duration of breaks between exercise bouts is short and recovery time is inadequate, it can lead to acute reversible microinjuries to the heart that can accumulate. Eventually, this leads to structural and functional remodeling (e.g., fibrosis, arrhythmias, etc.), particularly in the atria and right ventricle.

CTnT is found mainly in the fine myofilaments of myocardial fibers and is highly specific and sensitive for the diagnosis of myocardial ischemia. Serum cTnT concentrations are significantly elevated after marathon exercise, with reversible myocardial damage and transient cardiac dysfunction [38]. Amateur marathon runners are more likely to have elevated cTnT levels and transient myocardial injury compared to professionally trained runners. The results of this study indicate that the levels of myocardial biomarkers in the experimental and control groups showed similar trends of change after marathon running (i.e., increased after the race and did not recover after 24 h). Only cTnI in the experimental group returned to pre-race values at 24 h after the race. In contrast, in the control group, the cTnI level remained significantly elevated (*p* < 0.01) from pre-race to 24 h post-race. Thus, the control group showed a potential risk of myocardial injury. The general patterns of changes in cardiac biomarker levels in this study were consistent with the extensive literature on this topic, which shows transient increases in myocardial cell injury and wall stress parameters.

A sustained increase in cardiac output in the hours following a race leads to increased right atrial and ventricular wall tension and dilatation of these chambers, causing the release of cytosol biomarkers [7,8], without an actual breakdown of myocytes [9]. Low-level inexperienced runners participating in ultra-long-distance races (e.g., 118 km ultramarathons [39] and 24 h races [40]) also showed increased blood NT-proBNP levels. This may be because individuals who train less may have lower pulmonary artery systolic pressure during exercise compared to trained athletes, which may affect NT-proBNP release [9] and stimulate the release of cardiac biomarkers at exercise intensities above those to which the individual is accustomed [9]. For amateurs, especially those at lower levels, finishing the race may be more important than the finish time; hence, amateurs may choose a speed with which they are comfortable and familiar (i.e., training speed).

In this study, the analysis of serum cTnI, CK, CK-MB, LDH, and AST measured before, immediately after, and 24 h after the race in amateur marathon runners revealed elevated levels of myocardial injury markers in both the experimental and control groups compared to pre-race levels. The increases were more significant in the control group than in the experimental group. These findings suggest that there was myocardial injury in the control group, which caused elevated myocardial markers at the early stage levels that decreased with increased exercise levels. Furthermore, myocardial injury markers did not fully recover at 24 h after exercise, indicating persistent and more severe myocardial injury, especially in the control group, with lower exercise levels.

### 4.3. Strengths and Limitations

The major strengths of this study are the use of objectively measured cardiac morphology, function, and injury indicators in a laboratory setting and the investigation of differences in these outcomes between high- and low-level Chinese amateur distance runners. As many studies on this topic have relied on self-reported data, our study findings may provide meaningful and practical advice to runners of various levels for health promotion and disease prevention. Despite these strengths, this study also has several limitations that deserve our attention. First, the study participants were only from a region in south-central China, and the sample size was modest. Future studies with large-scale and more diverse samples are needed. Second, due to the nature of the design in this study, causal relationships among the study variables and athletic levels could not be determined. Thus, future longitudinal design is strongly recommended to further confirm the causality among athletic levels and cardiac morphology, function, and injury indicators in Chinese amateur endurance runners.

## 5. Conclusions

It is known that regular participation in physical activity has numerous physical, psychological, and mental health benefits for various populations and is thus highly recommended by the World Health Organization [41,42,43,44,45,46]. Promoting participation in physical activity has become a public health endeavor in the past decades. Among numerous physical activity programs, endurance and ultra-endurance events are becoming increasingly popular among amateur endurance runners. Extreme over-exercising is an issue that can lead to cardiac-associated exercise risks. This study investigated the effects of long-term endurance exercise on cardiac morphology, function, and injury indicators among amateur marathon runners. In summary, the findings suggest that high-level amateur marathon runners had greater heart volume, heart wall, and septal thickness compared to low-level marathon runners, most notably in heart volume. The performance of the higher-level marathon runners and the higher myocardial contractility were major factors in maintaining their greater ventricular diastolic amplitude and stroke volume. The practical implication of this finding is that in the future, amateur runners should be trained for a sufficient number of hours by professionals and reach at least National Athletic Grade 2 before running a marathon. In addition, the marathon damaged the hearts of both high-level and low-level amateur runners; however, runners with high athletic levels showed higher myocardial repair ability, with a greater regression in the levels of myocardial injury marker levels at 24 h post-race, whereas runners with lower athletic levels showed poorer myocardial injury repair ability. Thus, regular training sessions are warranted for amateur marathon runners, regardless of their athletic levels.

## Figures and Tables

**Table 1:** Baseline parameters of amateur marathon runners in Hengyang (mean ± standard deviation).

Parameter	Control Group (*n* = 18)	Experimental Group (*n* = 15)	*p*-Values
Age (years)	38 ± 7	35 ± 9	>0.05
Body mass index (kg/m^2^)	24 ± 3	23 ± 2	>0.05
Left ventricular mass index (g/m^2^)	118 ± 18.12	115 ± 28.26	>0.05
Blood pressure (mm Hg)			
Systolic	126 ± 18	123 ± 23	>0.05
Diastolic	81 ± 8	84 ± 6	>0.05
Basal heart rate (beats/min)	68 ± 5.21	58 ± 8.52	<0.05 *
Average weekly training distance (km)	35 ± 14.31	53 ± 17.61	<0.05 *
Duration of long-distance training (years)	9 ± 3.52	14 ± 3.17	<0.05 *
Number of marathons participated in	9 ± 6.44	12 ± 8.25	>0.05
Most recent marathon completion time (min)	293 ± 48	233 ± 48	<0.01 **

* *p* < 0.05, ** *p* < 0.01 compared to the control group.

**Table 2:** Characteristics of changes in morphological indicators before, immediately after, and 24 h after the marathon.

Indicator	Control Group (*n* = 18)			Experimental Group (*n* = 15)		
	Pre-Race	Immediate Post-Race Period	24 h Post-Race	Pre-Race	Immediate Post-Race Period	24 h Post-Race
EDV (mL)	101.63 ± 9.27	94.32 ± 11.49 ★★	99.98 ± 4.98	139.24 ± 9.56 **	115.83 ± 12.31 *★★	138.91 ± 15.46 **
ESV (mL)	34.51 ± 3.21	26.71 ± 4.01 ★★	30.34 ± 2.78	45.15 ± 4.52 **	24.89 ± 3.21 *★★	40.26 ± 4.82 **
IVSD (cm)	0.61 ± 0.21	0.63 ± 0.41	0.65 ± 0.29	0.87 ± 0.22 *	0.76 ± 0.19 *	0.83 ± 0.61
IVSS (cm)	1.16 ± 0.31	1.39 ± 0.51 ★★	1.23 ± 0.37	1.29 ± 0.53 *	1.36 ± 0.29	1.31 ± 0.94 **
LVPWD (cm)	0.86 ± 0.23	0.86 ± 0.33	0.86 ± 0.24	0.80 ± 0.12	0.87 ± 0.16	0.86 ± 0.19
LVPWS (cm)	0.86 ± 0.14	0.88 ± 0.22	0.87 ± 0.18	0.87 ± 0.26	0.89 ± 0.12	0.87 ± 0.19
LVPWM (cm)	0.86 ± 0.13	0.85 ± 0.12	0.85 ± 0.17	0.81 ± 0.11	0.87 ± 0.14 ★	0.87 ± 0.11

* *p* < 0.05, ** *p* < 0.01 compared to the control group for the particular indicator; ★ *p* < 0.05, ★★ *p* < 0.01 compared to the pre-race value for the particular group.

**Table 3:** Changes in cardiac functional indicators before, immediately after, and 24 h after the marathon race.

Indicator	Control Group (*n* = 18)			Experimental Group (*n* = 15)		
	Pre-Race	Immediately Post-Race	24 h Post-Race	Pre-Race	Immediately Post-Race	24 h Post-Race
SV (mL)	71.6 1 ± 9.79	72.71 ± 11.21	72.36 ± 16.56	90.31 ± 19.21 **	95.34 ± 9.98 **	90.63 ± 18.48 **
CO (L)	5.72 ± 10.21	13.31 ± 2.99 ★★	6.76 ± 8.91	6.21 ± 1.56	18.35 ± 2.75 ★★	9.76 ± 2.95 *★
EF (%)	70.12 ± 6.21	75.71 ± 7.84	71.84 ± 3.89	70.12 ± 3.38	81.54 ± 6.32 **★★	70.52 ± 5.24
%FS (%)	40.32 ± 4.91	52.12 ± 10.21 ★★	42.55 ± 7.71	39.52 ± 3.57	48.13 ± 5.93 **★	38.84 ± 6.24 **

Note: * *p* < 0.05, ** *p* < 0.01 compared to the control group; ★ *p* < 0.05, ★★ *p* < 0.01 compared to pre-race levels for the particular group.

**Table 4:** Changes in the levels of markers of myocardial injury before, immediately after, and 24 h after the marathon race.

	Control Group (*n* = 18)			Experimental Group (*n* = 15)		
	Pre-Race	Immediately Post-Race	24 h Post-Race	Pre-Race	Immediately Post-Race	24 h Post-Race
cTnI (ng/L)	0.10 ± 0.01	0.63 ± 0.22 ★★	0.27 ± 0.07 ★★	0.08 ± 0.00	0.43 ± 0.12 **★★	0.14 ± 0.07 **
CK (U/L)	87.65 ± 12.87	397.84 ± 24.61 ★★	247.37 ± 33.45 ★★	97.95 ± 24.33	257.97 ± 20.13 **★★	177.97 ± 23.18 **★★
CK-MB (U/L)	9.14 ± 3.57	57.65 ± 9.15 ★★	34.54 ± 8.48 ★★	8.31 ± 4.37	48.24 ± 7.46 **★★	26.15 ± 9.44 **★★
LDH (U/L)	157.64 ± 27.54	648.34 ± 42.56 ★★	314.78 ± 21.47 ★★	204.34 ± 18.46	547.23 ± 41.32 **★★	287.97 ± 35.08 **★
AST (U/L)	25.67 ± 6.34	58.79 ± 8.74 ★★	43.65 ± 7.46 ★★	24.31 ± 5.37	48.45 ± 8.78 **★★	30.73 ± 9.18 **★
NT-proBNP (ng/L)	143.67 ± 11.81	141.12 ± 19.91	141.83 ± 21.81	148.35 ± 13.61	150.71 ± 23.61	153.82 ± 21.41

** *p* < 0.01 compared to the control group; ★ *p* < 0.05, ★★ *p* < 0.01 compared to pre-race values.

## Data Availability

The data presented in this study are available on request from the corresponding author. The data are not publicly available due to the lack of an online server for data storage.
